# Lipidomic remodeling during mammalian preimplantation embryonic development

**DOI:** 10.1093/lifemeta/loae005

**Published:** 2024-02-28

**Authors:** Qingran Kong, Shaorong Gao

**Affiliations:** Zhejiang Provincial Key Laboratory of Medical Genetics, Key Laboratory of Laboratory Medicine, Ministry of Education, School of Laboratory Medicine and Life Sciences, Wenzhou Medical University, Wenzhou, Zhejiang 325035, China; Clinical and Translational Research Center of Shanghai First Maternity and Infant Hospital, Shanghai Key Laboratory of Signaling and Disease Research, Frontier Science Center for Stem Cell Research, School of Life Sciences and Technology, Tongji University, Shanghai 200092, China

**The dynamic changes in lipids during early embryonic development in mammals have not yet been comprehensively investigated. In a recent paper published in *Nature Cell Biology*, Zhang *et al*. reported the dynamic lipid landscapes during preimplantation embryonic development in mice and humans. They highlight the crucial role of lipid unsaturation in regulating embryogenesis.**

Mammalian life begins with the fusion of an oocyte and a sperm, and these two terminally differentiated germ cells are converted into a totipotent zygote. Zygote then undergoes a series of crucial biological events, including zygotic genome activation (ZGA), polarity establishment, initial cell-fate decision, and lineage-specific differentiation of the inner cell mass (ICM) and trophectoderm (TE), giving rise to a blastocyst. Cellular metabolism underlies all biological activities. Metabolic reprogramming is hardwired into the complex program of early mammalian embryo development, directly involving alterations in chromatin and DNA states. The significance of cellular metabolism in controlling preimplantation embryonic development was recognized several decades ago through the identification of the conditions that allow early embryos to grow outside of the oviduct [1]. Embryos at the early cleavage stage exist in a metabolically quiescent state, which requires pyruvate and lactate. Pyruvate is vital for development beyond the 2-cell stage and ZGA, facilitated by the selective translocation of key mitochondrial tricarboxylic acid (TCA) cycle proteins to the nucleus [2]. Our study has shown that lactate is also crucial for ZGA, leading to the establishment of histone H3 lysine 18 (H3K18) acetylation at the promoter regions of major ZGA genes in humans and mice [3]. Following ZGA, the embryo transitions into a highly oxidative state at the later stages, using glucose to support blastocyst development. A glucose-mediated signaling process plays a critical role in regulating the expression of transcription factors essential for TE differentiation [4].

The metabolism of mammalian early embryos has traditionally been studied using radiolabeled substrates in combination with inhibitors and exogenous substrates at specific stages. This approach is exemplified by the work of Sharpley *et al*., who demonstrated the metabolic plasticity during mouse preimplantation embryonic development [2]. However, the comprehensive understanding of the dynamic changes of metabolites and their associated metabolic pathways has been hindered by limitations in obtaining sufficient embryo samples. With the advancement in high-performance liquid chromatography coupled with mass spectrometry technology, research has begun to shed light on the metabolomic profile of early mammalian embryos. We have reported a metabolome profile of preimplantation embryos of mice from the zygote to blastocyst stages. Our findings have shown that the combination of the prominent metabolic cofactor nicotinamide adenine dinucleotide (NAD^+^) and the deacetylase sirtuin 1 (SIRT1) removes zygotic H3K27 acetylation (H3K27ac) in the late 2-cell stage for the exit of the minor ZGA, which is essential for mouse and human preimplantation embryonic development [1]. Additionally, Jin Zhang’s group has established the metabolomic profiling of mouse early embryos. Their work highlights the role of reciprocal changes in a pair of competitive metabolites (α-ketoglutarate (α-KG) and 2-hydroxyglutarate (2-HG)) in the dynamic erasure of histone methylation during blastocyst formation in mice [5]. In their most recent work, based on the optimized ultra-low-input metabolomics approach, Zhang *et al*. have established the dynamic lipid landscapes during mouse and human preimplantation embryonic development [6].

In this study, Zhang *et al*. mainly exhibited the dynamics of lipid composition, including phospholipids, sphingolipids, and neutral lipids. Phospholipids, the primary components of the cell membrane, play a crucial role in cell proliferation, including phosphatidylcholines (PCs), phosphatidylethanolamines (PEs), phosphatidylinositols (PIs), phosphatidylserines (PSs), and cardiolipins (CLs). The authors observed that PCs were the most abundant and displayed a bimodal distribution with higher levels at the oocyte and blastocyst stages. PEs exhibited a steady reduction in abundance from oocytes to blastocysts. PCs and PEs primarily reside in the outer and inner leaflets of the plasma membrane, respectively [7]. These results suggest that dynamic membrane remodeling occurs during preimplantation embryonic development. PI levels were the highest in oocytes and zygotes and gradually decreased in the 4-cell stage. PIs can function as signaling molecules, and inositol 1,4,5-trisphosphate can participate in the release of Ca^2+^ into the cytosol and promote oocyte activation. Thus, the dynamics of PIs align with their role in postfertilization oocyte activation. Most of the CLs were observed to enrich from the 4-cell stage. CLs are located in the inner membrane of mitochondria to maintain mitochondrial integrity and function in respiratory supercomplex formation, cristae biogenesis, and the TCA cycle. The increased CL levels coincide with the increased mitochondrial functions during preimplantation embryonic development. Sphingolipids, primarily consisting of sphingomyelins (SMs), ceramides (Cers), and glycosphingolipids, form specialized microdomains on the cell membrane and promote signal transduction. The authors showed that most SMs, which are essential cellular structure components for lipid rafts, were remarkably enriched at the blastocyst stage. This suggests that the formation of lipid rafts increases in the blastocysts to accommodate signal transduction molecules on the embryo cell surface. Neutral lipids are comprised of triacylglycerols (TAGs). TAGs mainly consist of long-chain fatty acids and glycerol and are the major components of lipid droplets (LDs) for energy storage, which is critical for preimplantation embryonic development [8]. The authors observed that the enrichments of TAGs with carbon chain lengths of 50–56 peaked at the 4-cell and 8-cell stages, whereas those with carbon chain lengths of 54–56 peaked at the blastocyst stage. This agrees with the results of neutral lipid-labeled methods showing that LDs accumulate at a later stage. The results support the increasing energy demand and cell proliferation during blastocyst development. The group also profiled the lipidome from the human 8-cell embryo stage to the blastocyst stage. The lipidomic profile exhibited a similar trend of changes, such as decreased PIs and PE levels, and increased CLs and TAGs levels, indicating that lipid remodeling is conserved in mouse and human preimplantation embryos.

Notably, lipidomic profiles revealed that the degree of unsaturation, especially in phospholipids, is a conserved characteristic between humans and mice as embryos progress to the blastocyst stage. The cell membrane, composed of lipid bilayers, exhibits a fluid-like property. The level of phospholipid unsaturation significantly influences membrane fluidity [9]. The authors discovered through fluorescence recovery after photobleaching analysis that plasma membrane fluidity decreased at the 2-cell stage and recovered at the blastocyst stage. Transcriptomic analysis of lipid metabolism genes revealed that the blastocyst was primarily enriched in the “biosynthesis of unsaturated fatty acids.” To investigate the production of unsaturated lipids enriched in blastocyst, the authors employed specific inhibitors and small interfering RNA (siRNA) to suppress lipid desaturases, such as stearoyl-CoA desaturase 1 (SCD1) and fatty acid desaturase 1 and 2 (FADS1/2), which produce monounsaturated fatty acids and polyunsaturated fatty acids, respectively. Significant decreases in the blastocyst formation rate and cavity area were observed in the fatty-acid-free potassium simplex optimization medium (KSOM). Moreover, these developmental abnormalities could be rescued by supplementing with oleic acid. Notably, a decrease in membrane fluidity was observed when SCD1 was inhibited, and this was also rescued by oleic acid supplementation. Transcriptomic analysis revealed that genes related to the apical plasma membrane and microtubule-based movement were downregulated, suggesting potential impairment in cytoskeleton organization during blastocyst development. To test this, the authors performed immunofluorescence of keratin 8 (K8) and keratin 18 (K18). They found that SCD1 inhibition disrupted the organized localization of K8 and K18, causing an obscured interface at the cell-cell boundary, and this effect could be rescued by oleic acid, demonstrating that lipid desaturases contribute to proper cytoskeleton organization in blastocyst development by producing unsaturated fatty acids. It is believed that during early embryo development, cytoskeleton components can link internal and external signals to promote apical domain formation. This establishes apical-basal polarity, facilitates blastocyst formation, and supports blastocyst implantation [10]. The authors showed that treatment with an SCD1 inhibitor led to a reduction in the enrichment of the phosphorylated apical protein ezrin and the basolateral protein E-cadherin in the blastocyst. Furthermore, the fluidity of ezrin and F-actin, whose lateral mobility is required for polarity establishment, was attenuated when SCD1 was inhibited. This effect could be rescued by oleic acid supplementation. As apical-basal polarity promotes TE cell specification [10], the authors also found that nuclear expression of the TE transcription factor Yes-associated protein was reduced in SCD1-inhibited blastocysts. More importantly, the uterus of pseudo-pregnant recipient mice, which were transferred with the SCD1-inhibited blastocysts, exhibited significantly fewer deciduae. Furthermore, the authors also showed that the inhibition of lipid desaturase affected the apical-basal polarity of mouse embryonic stem cells and trophoblast stem cells, as well as the efficiency of blastoid formation from expanded potential stem cells. Thus, the authors concluded that SCD1 is required for the establishment of apical-basal polarity to support blastocyst formation and implantation.

**Figure 1:**
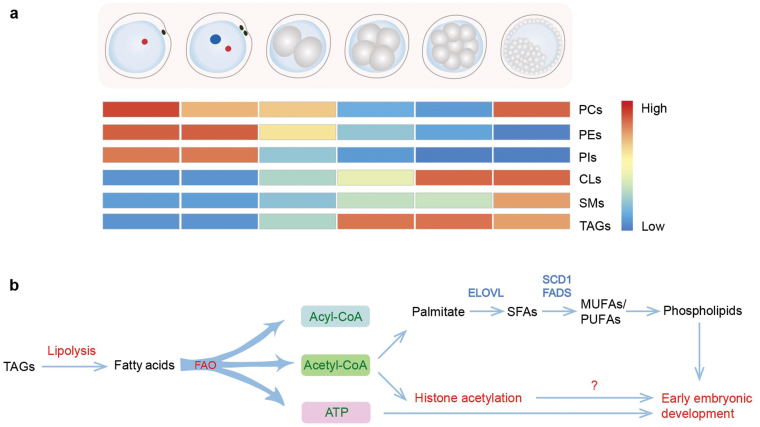
Schematic of lipid dynamics during preimplantation embryonic development (a) and lipid metabolism involved in the study by Zhang *et al*. (b) [6]. In the study, the authors highlight that lipid unsaturation by desaturases is required for blastocyst development. The remaining question is the role of unsaturated phospholipids generated by acetyl-CoA from FAO on embryogenesis. FAO is indispensable for the development of preimplantation embryos at late stages both *in vivo* and *in vitro*. The energy from FAO is not the key issue due to the wide resources of ATP, so acetyl-CoA derived from FAO is likely essential during the late stage. That is worthy to be further investigated. ELOVL: elongation of very long-chain fatty acids. MUFAs: monounsaturated fatty acids. PUFAs: polyunsaturated fatty acids. SFAs: saturated fatty acids.

Collectively, the study by Zhang *et al*. presents the first systematic examination of lipid dynamics during early embryonic development in humans and mice (Fig. 1a). It provides compelling evidence showing that lipid desaturases are required for *in vitro* blastocyst development and implantation (Fig. 1b). Like most innovative work, their findings generate many questions. For instance, how do unsaturated lipids function at the molecular level in cytoskeleton organization? Can the abnormalities be recapitulated when the SCD1 inhibitor is used specifically during the process of polarity establishment after the 8-cell stage? A critical question in the story is that the supplementation with oleic acid was not performed to rescue some SCD1 inhibition-induced abnormalities, such as the failure of blastocyst implantation and blastoid formation. This raises a question about the functions of SCD1 mediated-lipid unsaturation on polarity establishment during blastocyst development. More importantly, fatty acid β-oxidation (FAO) results in the shortening of fatty acids by two carbons per cycle, generating acetyl-CoA, which is a very efficient source for *de novo* synthesis of lipids. FAO plays a vital role in the development of preimplantation embryos. Oocytes with sufficient FAO lead to an improvement of subsequent embryo development in terms of the numbers of hatching blastocysts and the ICM:TE ratio. Given that, an interesting but tangential thought emerges from this work. What are the molecular mechanisms of FAO on preimplantation embryonic development? By synthesis of lipids, particularly unsaturated ones, or acetyl-CoA-mediated histone acetylation, such as H3K27ac, which is essential for ZGA program and preimplantation embryonic development in humans and mice [1] (Fig. 1b)? Nonetheless, this article has uncovered new and provocative biology relevant to lipid metabolism avenues for future investigation.
